# Reducing renal function assessment prior to platinum-based chemotherapy: a real-world evaluation

**DOI:** 10.2340/1651-226X.2024.23960

**Published:** 2024-04-10

**Authors:** Suzanne van der Gaag, Mariette Labots, Eleonora L. Swart, Mirjam Crul

**Affiliations:** a Department of Clinical Pharmacology and Pharmacy, Amsterdam UMC location Vrije Universiteit Amsterdam, Amsterdam, The Netherlands; b Department of Medical Oncology, Amsterdam UMC location Vrije Universiteit Amsterdam, Amsterdam, The Netherlands

**Keywords:** Platinum-based chemotherapy, carboplatin, cisplatin, renal function

## Abstract

**Background:**

Platinum-based chemotherapy, a widely used backbone of systemic cytotoxic anticancer treatment, is associated with nephrotoxicity. Currently, renal function is generally assessed prior to each administration of cisplatin or carboplatin, but there is no guideline regarding the frequency of renal function determination.

**Objective:**

The primary objective was to determine the median time to a clinically relevant dosage adjustment (>10%) due to change in renal function in patients treated with cisplatin and carboplatin. Secondly, variables influencing changes in renal function were assessed.

**Methods:**

We conducted a retrospective analysis of serial renal function assessments in platinum-treated patients with cancer in two academic medical centers, using a query to extract data from the electronic health records between 2017 and 2019.

**Results:**

In total, 512 patients receiving cisplatin and 628 patients receiving carboplatin were included. In total, 15% of all cisplatin-treated patients were found to have a renal function less than 60 mL/min at least once during treatment, with a median time to renal function decline of 67 days (range 5-96 days), which did not differ between treatment regimens. For carboplatin 21% of patients were found to have had a dosage variation of more than 10% at least once during treatment, with a median time-to-event period of 64 days (range 5-100 days).

**Interpretation:**

Dose adjustments during platinum-based chemotherapy resulting from renal function decline occur after a median time of ≥64 days. Our data provide substantiated guidance to recommend renal function assessment during platinum-based chemotherapy in clinically stable patients to once every 3 weeks.

## Background

Platinum derivatives are a widely used backbone of systemic cytotoxic anticancer treatment. Their use is associated with nephrotoxicity, although renal dysfunction occurs less frequently with carboplatin than with cisplatin, with an incidence of 10–15% and 20–30% respectively [1–6]. Nephrotoxicity is a broad term for a variety of kidney damage disorders like acute kidney injury (AKI), hypomagnesemia, hypocalcaemia, proteinuria, and chronic renal failure [5]. The primary mechanism behind nephrotoxicity in platinum derivatives is based on acute tubular necrosis by accumulation in the renal cells, affecting the proximal tubular cells and leading to AKI [7, 8]. Structural changes to the second-generation platinum derivatives such as oxaliplatin resulted in less accumulation and therefore less nephrotoxicity [5].

Currently, a recent assessment of renal function by an estimated glomerular filtration rate (eGFR) at baseline and during treatment is required for correct platinum dosage assignment. However, neither treatment guidelines nor clinical studies provide a definition of ‘recent’ in this context. In practice, renal function as measured by eGFR is currently determined before each administration, regardless of treatment interval.

The aim of this study is to gain insight into the predictability of decreased renal function when using the platinum derivatives carboplatin and cisplatin, and the influence of various variables on renal function decline. In addition, we would like to provide substantiated guidance for the frequency of renal function assessment in patients who are being treated with cisplatin or carboplatin.

## Materials and methods

### Setting and study population

A retrospective analysis of renal function assessments performed in patients with solid or hematological malignancies before and during platinum-based chemotherapy was conducted at Amsterdam UMC, location VUmc and location AMC, using data from January 2017 to January 2019. Data was collected from the electronic health records (Epic). Adults undergoing carboplatin and/or cisplatin treatment and for whom at least two creatinine determinations were available, including a pre-treatment assessment, were included. Several clinical variables potentially impacting renal function were also collected, including baseline renal function, gender, age, diagnosis, tumor stage, curative versus palliative intent, and treatment regimen. Oxaliplatin was omitted from this study, as renal function monitoring is not used to modify doses during treatment for this medicinal product. The Medical Ethics Review Committee at Amsterdam UMC, location VUmc, declared that the study was not subject to the Medical Research Involving Human Subjects Act (Non-WMO declaration 2019.313). Patient data were handled in accordance with privacy legislation.

### Clinically relevant decline in renal function and dosage adjustment

Prior to data analysis, clinically relevant renal function decline and platinum dosage adjustments were defined by clinicians of Amsterdam UMC. For cisplatin, a decrease in the eGFR to less than 60 mL/min was defined as a relevant change as this results in a dosage adjustment or in a switch to the less nephrotoxic carboplatin. For carboplatin, a dosage adjustment (increase or decrease) ≥10% was defined as clinically relevant. For both, an event was defined as the occurrence of a clinically relevant change.

### Stratification of dosing schedules

In order to discern between different dosing regimens, the dosages of cis- and carboplatin have been stratified. Cisplatin is subdivided into three dosing groups, aligning with the various dosing regimens. These groups for cisplatin are 0–20 mg/m^2^ for a weekly administration, 20–40 mg/m^2^ to represent a split-dose administration, and >40 mg/m^2^ for a three-weekly schedule. For carboplatin, a distinction is made at AUC (Area Under the Curve) < 4, suitable for low-dose carboplatin in a weekly schedule, and AUC ≥ 4, suitable for a high-dose carboplatin in a three-weekly schedule.

### Endpoints

The primary objective was to determine the median time to a clinically relevant dosage adjustment based on a change in renal function in patients receiving cisplatin and carboplatin treatment.

The secondary objective was to determine the influence of variables such as gender, age, diagnosis, tumor staging, treatment goal (curative or palliative), treatment regimen, and baseline renal function on dosage adjustments and the changes in renal function.

### Data analysis

All quantitative data were analyzed by SPSS (version 26.0.0.1 SPSS, Inc. Chicago, IL, USA). Descriptive analyses were used for baseline characteristics. Categorical variables were described in terms of numbers and percentages. Continuous variables for normally distributed data were described by the mean (standard deviation), while those for non-normally distributed data were described by the median (range).

Multivariate logistic regression analysis with generalized estimating equations (GEE) was used to determine the relevance of the different variables to changes in renal function during therapy with platinum derivatives. The multivariate Cox regression model used included age, gender, and dosage. A *p*-value of 0.05 was used for statistical significance.

## Results

A total of 1,140 patients with solid and hematological tumors were included: 512 treated with cisplatin and 628 treated with carboplatin. Patients from the cisplatin-group with a median age of 57 years received a median of three cycles with a median dosage of 50 mg/m^2^, and had a median baseline eGFR of 98.5 mL/min (Table 1). Patients from the carboplatin-group with a median age of 64 years received a median of four cycles of carboplatin with a median AUC of 4, and had a median baseline serum creatinine of 89.6 mL/min (Table 1).

**Table 1:** Basic characteristics of cisplatin group (*n* = 512) and carboplatin group (*n* = 628).

	Cisplatin (*n* = 512)	Carboplatin (*n* = 628)
Male, *n* (%)	264 (51.6)	307 (48.9)
Age (years), avg. ± SD	56.8 ± 14.3	64.4 ± 11.8
Treatment goal Curative Palliative Unknown	318 (62.1) 106 (20.7) 88 (17.2)	239 (38.1) 155 (24.7) 234 (37.3)
Number of cycles, median (range)	3 (1–14)	4 (1–8)
Baseline eGFR*, median (range)	98.5 (30–120)^$^	89.6 (24–120)^$^
Dosage, *n* (%) 0–20 mg/m^2^ (weekly dosing) 21–40 mg/m^2^ (split dose) > 40 mg/m^2^ (3-weekly dosing) AUC < 4 AUC ≥ 4	77 (15.2) 157 (30.9) 274 (53.9) N/A N/A	N/A N/A N/A 271 (43.2) 357 (56.8)
Pre-existing renal insufficiency, *n* (%)^#^	39 (7.6)	113 (18.0)

AUC: Area Under the Curve.

* eGFR calculated from measured creatinine, using CKD-EPI.

$ eGFR is capped at a maximum of 120 mL/min/1.73 m^2^.

# Renal insufficiency defined as a renal function < 60 mL/min.

### Cisplatin

**Figure 1:**
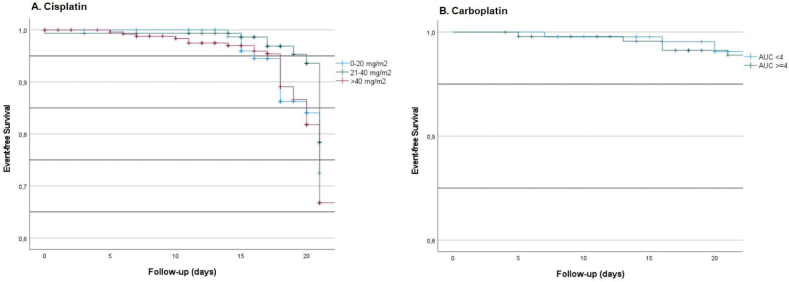
Event-free survival of cisplatin (A) and carboplatin (B) at 21 days.

Cisplatin was administered primarily for tumors of the head and neck (25%), cervix (20%), and lung (13%). Doses above 40 mg/m^2^ were administered in 52% of cases (Table 1). In total, 15% of all cisplatin-treated patients were found to have a renal function of less than 60 mL/min at least once during their treatment, which did not differ between treatment regimens. After 100 days of follow-up, 40% of patients experienced at least one event (a clinically relevant renal function decline to less than 60 mL/min), with a median time-to-event of 67 days (range 5–96 days). The event-free survival at 21 days is shown in Figure 1A, where >70% did not have a relevant renal function decline in 21 days. Lower doses showed less renal function decline compared to higher doses.

Men were found to be approximately twice as likely as women to experience an event (*p* = 0.012, Table 2). In addition, the probability of an event increased by 3.5% with each additional year of life (*p* < 0.001). Finally, it was notable that regimens in which cisplatin was combined with radiotherapy, were associated with a significantly higher probability of an event than cisplatin regimens that did not involve radiotherapy (*p* < 0.001). The significance of the individual variables was confirmed by multiple logistic regression analysis (Table 2).

**Table 2:** Influence of pre-specified variables on the endpoint, determined using Generalized Estimating Equations.

Variable	Cisplatin		Carboplatin	
	*p*	Hazard ratio	*p*	Hazard ratio
Age	< 0.001	1.061	< 0.001	1.026
Gender	0.011	2.163	0.188	1.179
Dosage	0.245	0.995	0.052	0.999
Tumor type	< 0.001	^#^	0.016	^#^
Treatment goal	0.378	0.764	0.156	0.793
Baseline eGFR	< 0.001	1.083	< 0.001	0.994
Cycle number	0.147	0.926	< 0.001	0.847
Interval between renal function assessment and administration	0.552	0.994	0.049	0.996

# depending on tumor type relative to reference tumor type (ref. cisplatin head neck, ref. carboplatin lung).

**Figure 2:**
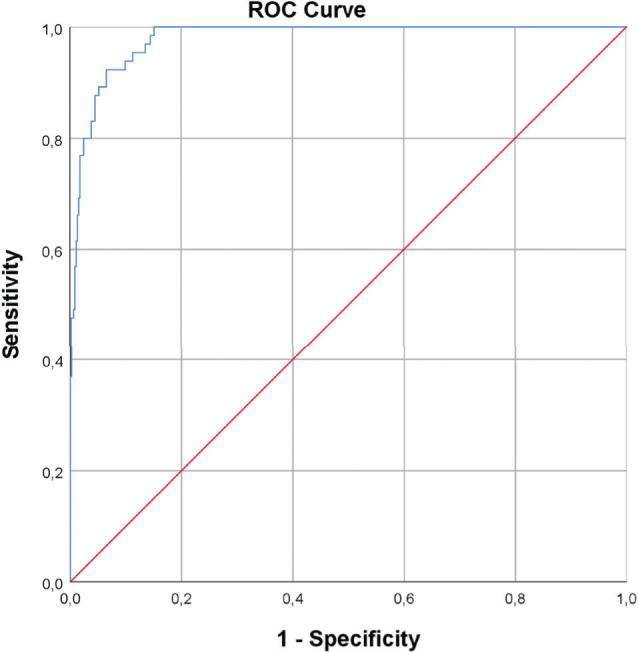
Receiver Operating Characteristic-curve for cisplatin’s prediction model.

In the case of cisplatin, a clinically relevant change in renal function was shown to be upfront highly predictable using a model including four variables (gender, age, creatinine at baseline, dosage), with a sensitivity of 93.9% and a specificity of 83.5% (Figure 2). These variables and their specific influence on renal toxicity were used to construct a prediction model, using the following formula:

$$Chance\;of\;event(\%)=\left(\frac{e^{-17.385+fact.Gender+0.1*Age+0.108*Creat+Dosing.cat}}{1+e^{-17.385+fact.Gender+0.1*Age+0.108*Creat+Dosing.cat}}\right)*100$$

*Factor for gender (fact.Gender): Male = 0, Female = 1.048, Creatinine (Creat), Dosing category (Dosing.cat): 0–20 mg/m^2^ = 0, 21–40 mg/m^2^ = 1, >40 mg/m^2^ = 2*.

### Carboplatin

Carboplatin was administered primarily for tumors of the lung (34%), esophagus (28%), and ovaries (12%), with a median AUC of 4 (range 1–11). The distribution between a low AUC (<4, weekly regimen) and a high AUC (≥4, 3-weekly regimen) was 43 and 57%, respectively. In total, 21% of all carboplatin-treated patients were found to have had a dosage variation of more than 10% at least once during their treatment, with a median time-to-event period of 64 days (range 5–100 days). The event-free survival at 21 days is shown in Figure 1B, where 97% of patients did not have any event within 21 days.

Increasing age and lower baseline renal function significantly increased the probability of a clinically relevant dosage adjustment (*p* < 0.001). Interestingly, an increase in the number of cycles was associated with reduced probability of an event by about 15% per cycle (*p* < 0.001). The significance of the individual variables was confirmed by multiple logistic regression analysis.

**Figure 3:**
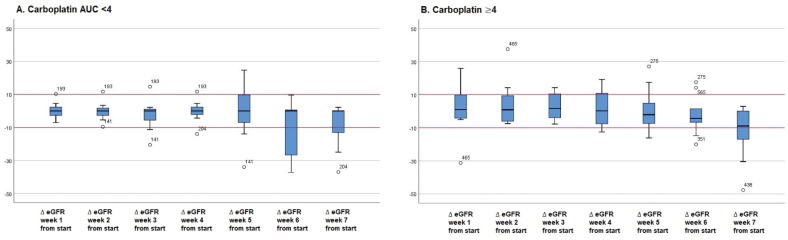
Change in eGFR relative to baseline for carboplatin dosed at AUC < 4 (A) and ≥4 (B).

Analyzing eGFR change relative to baseline, a low AUC was found to involve less variation than a high AUC (Figure 3). Unlike cisplatin, a change of renal function as a result of carboplatin treatment could not be predicted by a model.

## Discussion

In our analysis of 1,140 patients, renal function decline requiring dosage adjustment of cis- and carboplatin-based chemotherapy occurred with a median time to event of 67 days for cisplatin and 64 days for carboplatin, regardless of dose intensity and schedule. While the interval for renal function assessment could potentially be extended, it is imperative to underscore the necessity of timely clinical evaluation to monitor for disease progression, which should not be deferred under any circumstances. Our study demonstrates that the extension of renal function assessment is feasible; however, its practical applicability is primarily pertinent to split-dose and weekly treatment regimens. For patients on three-weekly schedules, clinical assessment, including renal function evaluation, is conducted prior to each subsequent administration. For cisplatin, higher doses were associated with earlier occurrence of an event. For cisplatin a prediction model was constructed to predict the likelihood (%) of an event occurring in the coming 60 days. Most likely resulting from AUC-based dosing and therefore a large interpatient variability in dosing, such a prediction could not be made for the less nephrotoxic carboplatin.

In addition, there are multiple strategies available to reduce the toxicity of cisplatin. Hydration therapy stands as a cornerstone, aiding in the maintenance of renal function by promoting urine flow and thereby preventing cisplatin accumulation in the kidneys. Concurrently, management of magnesium levels helps mitigate cisplatin-induced nephrotoxicity, as magnesium supplementation can counteract renal tubular damage [9–11]. Employing split dosing strategies, wherein the total cisplatin dose is administered over several days instead of a single bolus, allows for better tolerability and reduced nephrotoxicity without compromising therapeutic efficacy.

A number of variables that influence the nephrotoxicity of platinum derivatives emerged from our multivariate logistic regression analysis, which include age, gender, dosage, baseline eGFR and number of cycles. The influence of age on the nephrotoxicity of platinum derivatives was recently described in a systematic review by Duan et al. [12] The overall increase in risk of nephrotoxicity induced by platinum derivatives was 43% in patients with a high age compared to young patients (*p* < 0.001), even though dose reductions were observed more frequently in the elderly. The risk of nephrotoxicity increasing with age was more prone in the cisplatin-treated patients compared to carboplatin-treated patients, namely 42 and 22% respectively. The mechanism behind the increased renal toxicity with increasing age includes a decrease in the size and number of nephrons, which results in greater susceptibility to nephrotoxicity [12]. The probability of an event increased by 3.5% with each additional year of life in our study, which could be comparable to the data presented by Duan et al, as 10–15 years of increase in age leads to the roughly 40% increase in nephrotoxicity.

With regard to gender, in 2017 a retrospective analysis was conducted on the gender differences in cisplatin-induced nephrotoxicity [13]. A survival analysis showed that women had a significant higher risk of development of kidney injury (*p* = 0.045), but after adjustment for confounders like hypertension and nephrotoxic co-medication, the risk became non-significant [13]. Our analysis found a higher probability of experiencing renal function change requiring dosage adjustment in men compared to women, as opposed to the non-significant increase in women in the retrospective analysis by Chen et al.

Finally, a prospective study by Ben Ayed et al. included 150 patients treated with cisplatin for various tumor types, mostly head neck cancer. Relevant variables significantly influencing the development of nephrotoxicity were age, type of cancer, chemotherapy regimens, and a cumulative dose of cisplatin [14]. All these variables are similar to the significant variables we found in our multivariate logistic regression analysis. For carboplatin, a small study of 10 patients with proven lung cancer found that eGFR decreased after multiple cycles of carboplatin. This supports the cumulative effect of renal toxicity after multiple cycles [15].

We included real world data from the Amsterdam UMC, which involved >1,100 patients treated with platinum compounds in different schedules, ranging from low to high nephrotoxicity risk. As there is currently no guideline for determining what constitutes a ‘recent’ measurement of renal function, the results of this study provide substantiated guidance for a renal function assessment, which has already been implemented in the Netherlands.

There were also some limitations to this study. Firstly, the retrospective nature of the study, where as a result, not all variables could be fully assessed. Comorbidities such as hypertension or diabetes, smoking status, (nephrotoxic) co-medication or relation with chemotherapy toxicity nor other factors than the pre-specified variables were not available for this analysis. Additionally, the limited scope for assessing all variables with the current sample size and the potential for heterogeneity among patients could potentially lead to outcome bias. Moreover, only the Amsterdam UMC participated, which may limit its external validity. Furthermore, discontinuation due to toxicity of platinum-based therapy can occur, which is not determined in this study. However, any discontinuations would not significantly impact the study outcomes, as patients would still undergo renal function assessment. Lastly, the definition of clinically relevant renal function alteration followed local procedures in consultation with the clinicians of Amsterdam UMC, in absence of guidelines and literature.

Some recommendations can be made for further research. Firstly, a multicentre database could be developed for external validation of the dataset and the prediction model. In addition, this would allow analysis of the influence of comorbidity and co-medication.

In conclusion, renal function alterations require dose adjustments during platinum-based chemotherapy. Since clinically relevant changes (events) can have a significant impact on patients’ safety and outcome, the recommendation for renal function assessment should be more frequent than the median time to event. Our data provide substantiated guidance to recommend renal function assessment during platinum-based chemotherapy in clinically stable patients to once every 3 weeks. This will not change the frequency of renal function assessment in patients treated with high-dose, three-weekly chemotherapy schedules with or without radiation therapy. However, renal function may be assessed less frequently for the low-dose schedules administered weekly or biweekly. Reducing the frequency of renal function assessment to three-weekly intervals would save many creatinine determinations per year and can increase efficiency in manufacturing and release of infusions for administration. The efficiency is improved by reducing the need to wait for lab results to set the final dosage and start manufacturing, which now occurs less frequently.

## Data Availability

The data generated during the study are not publicly available due to regulations on personal data protection but are available from the corresponding author upon reasonable request.
